# Secondary Metabolites Isolated from Chilean Marine Algae: A Review

**DOI:** 10.3390/md20050337

**Published:** 2022-05-23

**Authors:** Dioni Arrieche, Héctor Carrasco, Andrés F. Olea, Luis Espinoza, Aurelio San-Martín, Lautaro Taborga

**Affiliations:** 1 Laboratorio de Productos Naturales, Departamento de Química, Universidad Técnica Federico Santa María, Avenida España 1680, Valparaíso 2340000, CP, Chile; dioniarrieche@gmail.com (D.A.); luis.espinozac@usm.cl (L.E.); 2 Grupo QBAB, Instituto de Ciencias Químicas Aplicadas, Facultad de Ingeniería, Universidad Autónoma de Chile, Llano Subercaseaux 2801, Santiago 8900000, CP, Chile; hector.carrasco@uatotonoma.cl (H.C.); andres.olea@uautonoma.cl (A.F.O.); 3 Departamento de Ciencias y Recursos Naturales, Facultad de Ciencias Naturales, Universidad de Magallanes, Avenida Bulnes 01855, Punta Arenas 6200112, CP, Chile

**Keywords:** marine natural products, secondary metabolites, Chilean algae, biological activities, biosynthesis, structure elucidation

## Abstract

Chile is in the extreme southwestern part of America, and it has an extreme length, of approximately 4300 km that increases to 8000 km considering the Chilean Antarctic Territory. Despite the large extent of its coastal territory and the diversity of geographic environments and climates associated with Chilean coasts, the research on marine resources in Chile has been rather scarce. From marine organisms found in Chilean coastal waters, algae have been the most studied, since they contain a wide range of interesting secondary metabolites that have some structural traits that make them unique and uncharacteristic. Thus, a wide structural variety of natural products including terpenoids (monoterpenes, sesquiterpenes, diterpenes, and meroterpenoids), furanones, and C_15_-acetogenins have been isolated and identified. This review describes the existing literature on bioprospecting and exploration of secondary metabolites from Chilean coasts.

## 1. Introduction

Marine natural products have served as a rich source of new bioactive agents [1,2,3]. The diversity of marine habitats and unique sea environmental conditions have enabled marine organisms to develop mostly untapped sources of potential drugs with superior chemical novelty [4]. During the last decades, much effort has been dedicated to isolating and identifying new compounds from marine organisms, with the interesting outcome that many of these derivatives exhibited biological activities [5,6,7,8,9,10]. Algae are one of the simplest organisms containing chlorophyll, and, therefore, are found in almost every place where there is light to perform photosynthesis, namely in seas, lakes, rivers, animals and plants (as symbiotic species) [11]. They can be found as colonies of single-celled or multicellular organisms, and in some cases collaborating as simple tissues. Consequently, their size varies from 3–10 µm (unicellular algae) to 70 m long, i.e., giant kelp species growing up to 50 cm per day. Algae are classified into two major groups: microalgae, found both in benthic environments (littoral) and in the ocean (phytoplankton), and macroalgae (marine algae) red, brown and green algae, established in the littoral zone [12]. Phytoplankton comprise organisms such as diatoms (Bacillariophyta), dinoflagellates (Dinophyta), green and yellow-brown flagellates (Chlorophyta; Prasinophyta; Prymnesiophyta, Cryptophyta, and Rhaphidiophyta) and blue-green algae (Cyanophyta). These photosynthetic organisms play an important role in the productivity of oceans and are at the base of the food chain [11].

Marine macroalgae have been used in a number of important areas including the food industry [13], agriculture [14] and as raw materials on third-generation bioplastics [15]. However, despite their extended use for decades in traditional medicinal therapies, and the huge number of bioactive compounds that have been extracted and identified, algae are still underrepresented in the pharmaceutical industry. For example, compounds isolated from seaweeds showed interesting biological activities such as: antiprotozoal [16], antimicrobial [17,18,19], antifouling [20,21], anticancer [22], antileishmanial [23] and anti-inflammatory properties [24]. Between these compounds are: terpenoids, sterols, phenols, peptides, polysaccharides, acrylic acid, vitamins, proteins, heterocyclic compounds, chlorophyllides, halogenated ketones and alkanes as well as cyclic polysulfides [25]. From the large variety of metabolites isolated from algae, the most abundant compounds are terpenes, including monoterpenes, sesquiterpenes and diterpenes. These compounds, which are formed by different numbers of isoprene units (2-methylbuta-1,3-diene), have been found in volatile oils of terrestrial plants as well as in seaweed [26,27]. All of them possess great potential for further development in pharmacological applications [28].

**Figure 1:**
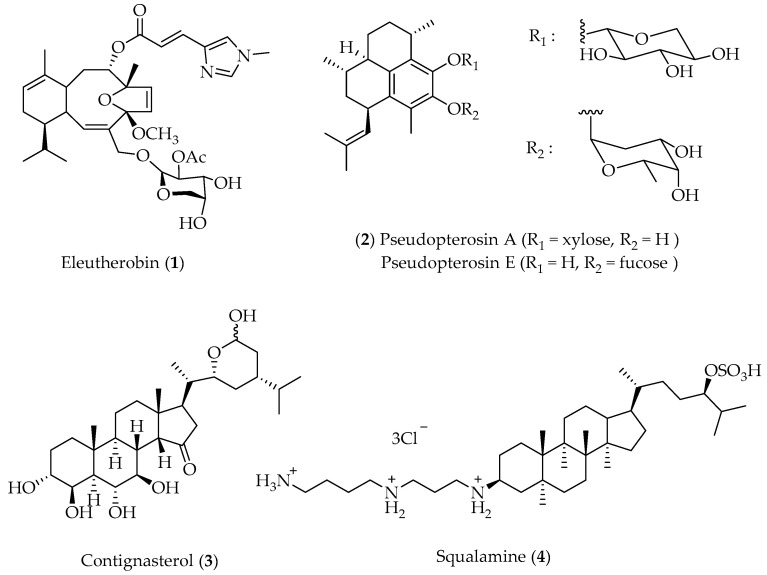
Terpenoid drug leads.

The biological importance of marine terpenes, as illustrated by their ecological role, may also be exploited in terms of their pharmacology. From this point of view, several biologically active terpenoids have been reported with biomedical potential, and some of them are already in preclinical or clinical development [29]. Eleutherobin (**1**) (Figure 1) originates from a soft coral of the genus *Eleutherobia* collected from Australian waters [30] and has been re-isolated along with congeners from another octocoral from the Caribbean [31], which can be maintained by aquaculture [32]. In preclinical experiments, eleutherobin has been employed as stabilizer of microtubuline, and it competes for the paclitaxel binding site on the microtubule polymer [33]. The anti-inflammatory pseudopterosins A–E (**2**) (Figure 1) are diterpene glycosides characterized by the presence of an amphilectane type skeleton. They were obtained from the gorgonian coral *Pseuodopterogorgia elisabethae* by Fenical’s group in the late 1980s [4,34]. Marine organisms, mainly sponges, contain unusual sterols such as contignasterol (**3**) (Figure 1), isolated from *Petrosia contignata* [35,36,37,38,39]. Contignasterol and its derivatives exhibit anti-inflammatory effects. Squalamine (**4**) (Figure 1) is a water-soluble cationic amino sterol occurring in the liver and stomach tissues of *Squalus acanthias*. The structure was published in 1993 and initially, it was reported as a potent antimicrobial agent with antibacterial, antifungal and anti-protozoic properties [40,41]. Subsequent studies demonstrated that this compound inhibits angiogenesis and tumor growth in various models [42], making it a good candidate for drug development as an innovative anticancer agent. Thus, a large quantity of terpenic compounds with important biological activity, isolated from different marine organisms, have been described in the literature [43].

It is worth noting that the reason why seaweeds produce such a vast spectrum of secondary metabolites is because they live in nonfriendly environments, and, therefore, they are forced to synthesize protective compounds and to develop protective mechanisms [44]. Abiotic stresses to which algae are exposed include rapid fluctuations in light intensity, temperature, osmotic stress and desiccation, which induce the formation of free radicals and oxidizing agents leading to photodynamic damage [45].

The benthic habitat maps of continental Chile show special topographic characteristics that have joined with coastal currents and winds to enhance the spread of algae, and consequently, around 440 species have been identified [46]. Therefore, the study of Chilean algae is a challenging task, and they are probably a unique source of new compounds with interesting biological activities [47]. Thus, in this work we present a review of the literature on terpenes, C_15_-acetogenins, and furanones as secondary metabolites isolated from Chilean marine algae. Scifinder databases as well as the repositories of the Pontificia Universidad Católica de Chile and Universidad Técnica Federico Santa María were used to search reports published from 1967 to the present. Regarding the search methods, these involved filtering by authors, e.g., Blunt, J.W.; San-Martín, A.; Darias, J.; Silva, M.; Norte, M., as well as by keywords, e.g., secondary metabolites, brown algae, and red algae, to name a few. The search criteria focused on algae obtained from Chilean coasts and the South Shetland Islands and reports of novel marine natural products. The latter were spectroscopically characterized and present interesting biological and pharmaceutical properties. Reports involving vegetable extracts or primary metabolites were omitted. If some contributions (works or results) were omitted, it was due to an unintended error that we deeply regret.

## 2. Secondary Metabolites Isolated from Chilean Algae

### 2.1. Terpenoids

**Figure 2:**
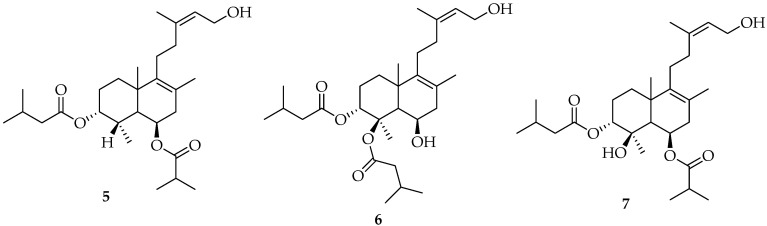
Terpenoids isolated from *Trimusculus peruvianus*.

Chilean waters and coasts have turned out to be a fertile source of a variety of marine organisms from which new terpenes have been obtained. For instance, the terpenes shown in Figure 2 were isolated and identified from *Trimusculus peruvianus*, a marine mollusk collected near Antofagasta coast, Chile. Compound **5**, identified as a new terpene, and previously reported compounds, **6** and **7** were assayed for their toxicity against *Artemia saline* [1,48,49,50]. 

#### 2.1.1. Structural Features of Marine Terpenes

**Figure 3:**
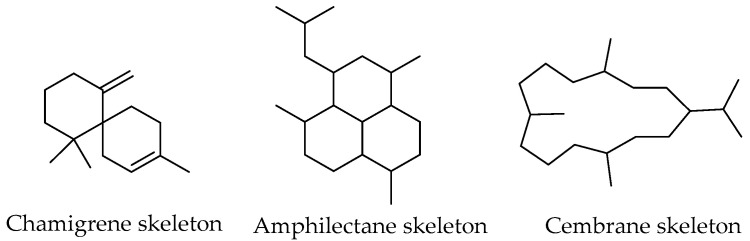
Common skeletons of terpenes isolated from marine organisms.

Some marine terpenes are known to have unique structural features. For instance, chamigrene [51], amphilectane [52], and cembrane [53] (Figure 3) exhibit unusual structures as well as uncommon functionalities, such as dichloroimine, isonitrile, isocyanate, isothiocyanate, and halogenated functions that are predominantly found in marine organisms. However, these functional groups are not exclusively marine [43]. 

Algae are frequently found in marine habitats, where they are exposed to environmental microorganisms. However, algae survive in these hostile environments, probably due to an inherently available chemical defense mechanisms. Therefore, many novel terpenes, such as monoterpenes [54], sesquiterpenes [55,56], diterpenes [57,58], meroterpenoids [59], and steroids [60,61], have been normally isolated from different seaweeds [62]. Hence, this review will be focused on terpenes isolated from marine algae collected in Chilean coasts, classified according to the number of carbon atoms present in their structural nuclei, and on their biological activities.

#### 2.1.2. Monoterpenes

Monoterpenes with multiple halogen substitution and uncommon carbon ring structures have been obtained mainly from red algae (Rhodophyta), and from green and brown algae, as well. They can be linear or cyclic (even heterocyclic) compounds [62].

##### Linear Polyhydroxylated Monoterpenes

**Figure 4:**
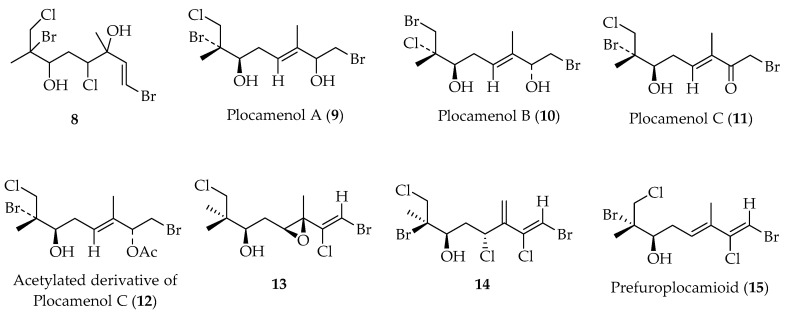
Linear polyhydroxylated monoterpenes isolated from Chilean algae.

Linear polyhalogenated monoterpene (**8)** and three plocamenols A–C (**9**–**11**) (Figure 4), are new compounds that were isolated from P. cartilagineum [63,64].

Compound **10** contains a terminal bromohydrin, whereas **11** is the corresponding keto derivative. To verify the correct HMBC correlations, **11** was acetylated using acetic anhydride in pyridine, leading to compound **12** [64]. Compounds **9** and **10**, along with costatol, are the first reported metabolites having a double bond conjugate bromohydrin [64]. Additionally, empirical rules based on ^13^C and ^1^H NMR spectroscopic analysis have been proposed to determine the regiochemistry and geometry of the 1,2-bromochloro vinyl portion. This is extremely relevant since it can be applied to any compound of natural or synthetic origin containing this functionality [65].

This structural feature was also found in two novel monoterpenes, **13** and **14**, (Figure 4), isolated from *P. cartilagineum*. The effect of γ-substituents on chemical shifts of C-1 and H-1 of the 1,2-dihalo vinylic portion was observed and used to validate the previously reported empirical rules for determining regio- and stereochemistry of substituted vicinal vinyl dihalide [66]. 

Prefuroplocamioid (**15**) (Figure 4) was isolated from *P. cartilagineum*, collected along the coast of Chile. This compound has been considered as a precursor of furoplocamioids (see below, compounds **34** and **35**) [65], suggesting that biosynthesis of the 1,2-bromochloro vinyl system occurs previously to oxetane ring formation of furoplocamioids.

**Figure 5:**
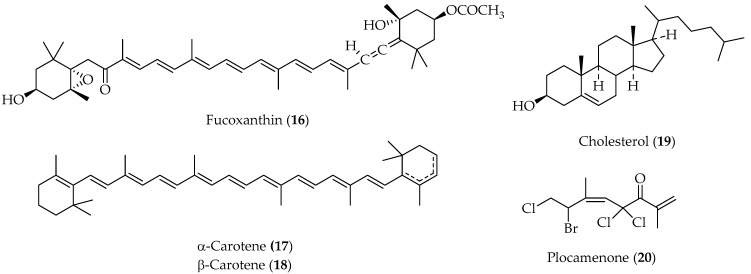
Secondary metabolites isolated from Chilean algae species *Ceramium rubrum*.

Several known compounds, including fucoxanthin (**16**), α and β-carotene (**17** and **18**), cholesterol (**19**) and plocamenone (**20**) (Figure 5) were isolated from the red algae, *Ceramium rubrum*, and spectroscopically characterized. The halogenated monoterpene **20** had been previously isolated and identified from the red algae *Plocamium* sp. [67].

##### Cyclohexane Polyhalogenated Monocyclic Monoterpenes

**Figure 6:**
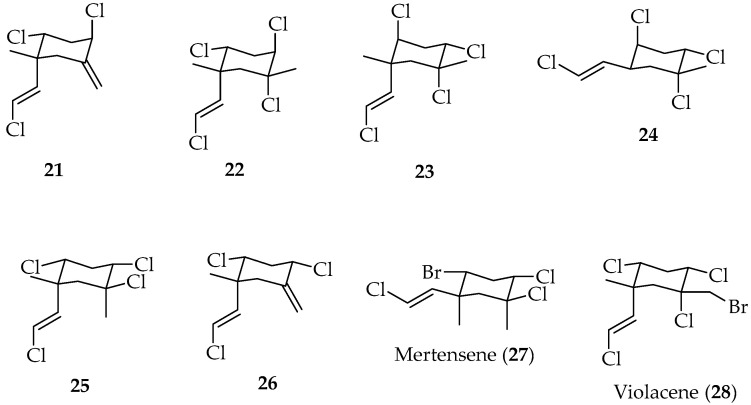
Polyhalogenated monocyclic monoterpenes, isolated from Chilean algae.

Polyhalogenated monocyclic monoterpenes, **21**–**28** (see Figure 6), were identified from red algae *Plocamium cartilagineum* collected at different points on the central Chilean coast (La Herradura (IV Region), Montemar and El Tabo (V Region), La Boca and Punta de Perros (VI Region), and Pumillahue (Chiloé, X Region)). Mertensene (**27**) and violacene (**28**), two brominated compounds, were identified spectroscopically by comparison with authentic samples [68].

A study carried out with samples of *P. cartilagineum*, collected in two different geographical areas (Quintay (V Region) and La Boca (VI Region)) showed very interesting results. The plant materials, divided into carposporophyte, tetraspomphyte and gametophyte-bearing plants, exhibited the same qualitative chemical composition but important quantitative differences for these three reproductive phases. Depending on their capacity to incorporate bromine, the collections were classified as α chemotype, terpenes with no bromine, and β chemotype, brominated monoterpenes. Compounds **21**, **22** and **26** were identified only in La Boca samples, whereas compounds **27** and **28** were characteristic of Quintay samples, and compounds **23**–**25** (Figure 6) were common to both collections [69]. The fungicide and insecticide/acaricide activities of several isolated derivates were determined; **28** showed the most potent insecticide activity among the compounds tested, mainly against *Macrosteles facifrons* [70].

Halogenated monoterpenes, **21**–**25** (Figure 6), were also isolated from *Shottera nicaensis*, which was collected intertidally at La Boca (VI Region), Chile. The total amount of compounds isolated from *S. nicaensis* was one order of magnitude lower than that obtained from *P. cartilagineum*, i.e., 0.04% and 0.5% dry weight, respectively. Even though for both algae the mixture composition turned out to be identical, small changes in the relative composition were observed. Both algae grow together, but their morphology and taxonomy are so different that it is almost impossible to mix them up. Even more unlikely is the idea that algae belonging to two different families have a common enzymatic system that allows the elaboration of identical compounds of almost the same composition. These facts seem to agree with Crew’s hypothesis about the algae *Microcladia* and *Shottera* growing in association with *Plocamium*, i.e., *Microcladia* and *Shottera* are able to concentrate the halogenated metabolites produced by *Plocamium* algae [71].

**Figure 7:**
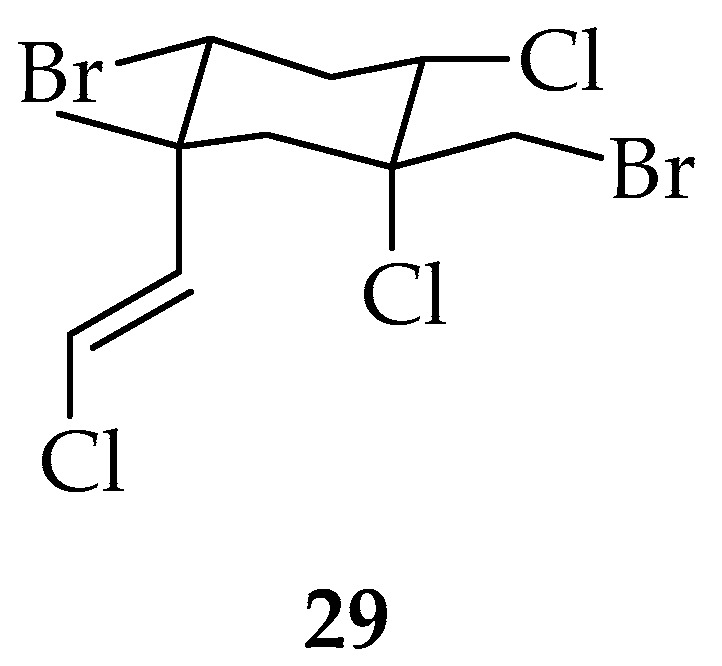
Polyhalogenated monocyclic monoterpene, obtained from endemic Antarctic species *Pantoneura plocamioides* and *P. cartilaginuem* L. (Dixon).

Finally, compound **29** (Figure 7) was isolated from the endemic Antarctic species *Pantoneura plocamioides* and *P. cartilaginuem* L. (Dixon), marine algae with a wide geographic distribution [72].

Compound **28** exhibited the greatest insecticidal activity, while **27** showed a moderate activity against Aphis fabae [73].

##### Tetrahydropyran Monoterpenes

**Figure 8:**
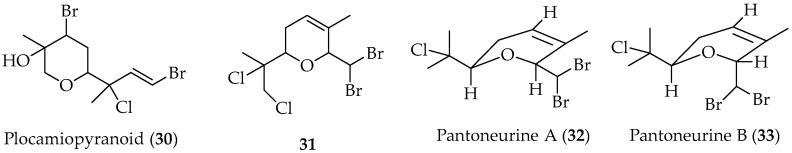
Tetrahydropyran monoterpenes isolated from Chilean algae.

Four new tetrahydropyran monoterpenes, **30**–**33** (Figure 8), have been isolated, and their structures and relative stereochemistry were determined using spectroscopic evidence [74]. Compounds **30** and **31** were obtained from *P. cartilagineum*, collected in El Yeco (V Region, Chile), whereas **32** and **33** were isolated from *P. plocamioides*, collected off King George Island (South Shetland, Antarctic).

##### Tetrahydrofuran Monoterpenes

**Figure 9:**
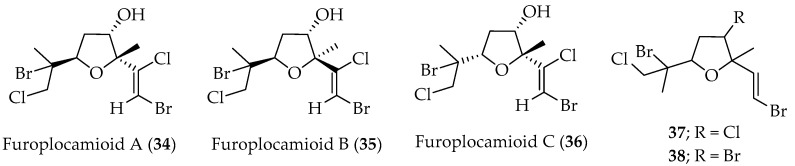
Tetrahydrofuran monoterpenes isolated from Chilean algae.

Tetrahydrofuran monoterpenes carrying non-common functional substituents, i.e., chloro or bromo vinyl groups, were identified in samples of *P. cartilagineum* collected off the central coast (V Region) of Chile. These compounds, **34**–**36** (Figure 9), are closely related to pantofuranoids obtained from the endemic Antarctic algae *P. plocamioides*, which indicates a close relationship between these species. The relative stereochemistry of these compounds was determined by spectroscopic experiments and molecular mechanics (MM2) calculations [75].

Two new related tetrahydrofuran halogenated monoterpenes, **37** and **38** (Figure 9), were isolated from *P. cartilagenium*, collected at El Quisco, in the V Region of Chile. Structural elucidation of these compounds has been reported [63] and indicates the presence of unusual vicinal vinyl dihalide, like that observed in furoplocamioids (A–C) (**34**–**36**).

Antifeedant activities of halogenated monoterpenes, i.e., **21**, **22**, **24**, **27**, **28**, **31**, **32**–**34**, **36**, **37**, and **39**, were tested against *Myzus persicae*, *Leptinotarsa decemlineata* and *Ropalosiphum padi*. It is worth noting that none of these derivatives showed phytotoxic effects [72].

In the same line, compounds **21**, **22**, **24**, **27**, **28**, **31**, and **34** have been tested for their cytotoxic activity on tumor cell lines CT26, SW480, HeLa and SkMel28 with several multidrug resistance mechanisms, and on mammalian non-tumor cell line CHO (Chinese hamster ovary cells). Results showed that compound **31** presents selective activity against SW480 and HeLa cells. An analysis of cellular extracts posterior to incubation with the assayed compounds and rotenone (positive uptake control) showed intracellular accumulation of **22**, **27, 31** and **34** [76].

On the other hand, the effect of photon flux density (PFD) and temperature on the relative growth rate (RGR) of *P. cartilagineum* and formation of three halogenated monoterpenes, **22**, **27** and **28**, has been assessed [77].

#### 2.1.3. Sesquiterpenes

Sesquiterpenes isolated from seaweeds can be classified, according to their carbon skeletons, into the following groups: laurene, chamigrane, brasilane, bisabolene, cuparane, and others [62]. Additionally, sesquiterpenes isolated from red algae are characterized by an elevated number of halogenated substitutions. These compounds play important roles, such as to defend algae against predators, fouling organisms and pathogens, as well as reproduction and protection against UV radiation, and serve as allelopathic agents.

*Laurencia* (Rhodophyceae) is the marine macroalgae genus that represents the most important source of sesquiterpenes. The reasons for this are, in the first place, that algae belonging to this genus are extremely widespread in the world, mainly from tropical and subtropical regions; second, they present a notable ability to biosynthesize a diversity of structurally different sesquiterpenes with new skeletons, such as (seco)- or (9,10-friedo)-chamigrane, (cyclo) perforane, guimarane, and poitane. Brown algae and green algae also partly contribute to marine sesquiterpenes. However, the presence of halogenated compounds is very unusual.

##### Chamigrene Sesquiterpenes

**Figure 10:**
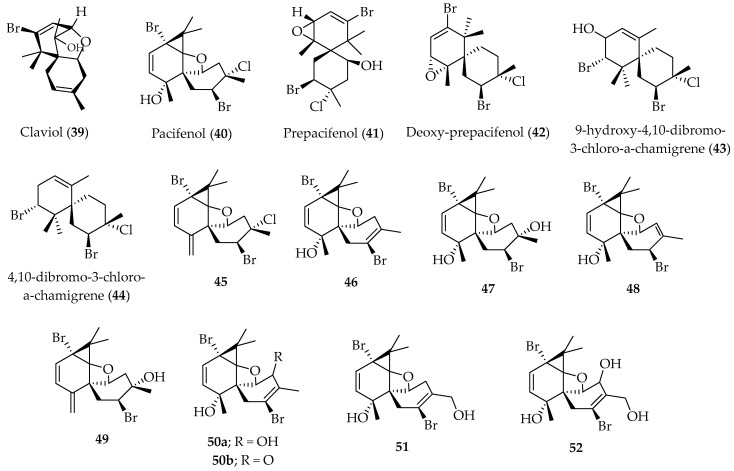
Claviol (**39**) and sesquiterpenes with chamigrene skeleton isolated from Chilean red alga *Laurencia claviformis*.

Claviol (**39**) and sesquiterpenes with the chamigrene skeleton, namely pacifenol (**40**), prepacifenol (**41**), deoxy-prepacifenol (**42**), 9-hydroxy-4,10-dibromo-3-chloro-α-chamigrene (**43**), and 4,10-dibromo-3-chloro-α-chamigrene (**44**), have been isolated from the red alga *Laurencia claviformis*, an endemic Easter Island species (see Figure 10) [78].

All of these compounds were assayed for inhibition of cytokinesis in the sea urchin *Tetrapygus niger* embryos, and **43** was identified as the most active compound. However, its activity could be considered mild as compared to that shown by stypoldione, another active marine compound with ED_50_ = 1.1 µg/mL. Nevertheless, this test seems to be a reasonable prescreen to determine which substances merit further evaluation for antineoplastic properties [78].

Finally, microbial transformation of pacifenol (**40**), and two semisynthetic derivatives, **45** and **46** (Figure 10), by *Aspergillus níger*, *Gibberella fujikuroi* and *Mucor plumbeus*, yielded new hydroxylated derivatives, **47**–**52** (Figure 10) [79].

#### 2.1.4. Diterpenes

Diterpenoids are found in higher plants, insects, fungi, and marine organisms. Several of these compounds present antimicrobial, antitumor, cytotoxic, anti-inflammatory, antifungal, molluscicide, antifeedant, and antifouling activities [43]. With more than 40 species reported, the genus *Dictyota* has been presented as a powerful resource for diterpenic compounds with novel chemical structures. Cyclic diterpenes are produced by many members of this genus, just like typical diterpenes with a 6-methyl-5-hepten-2-yl side chain A. Three types of main carbon skeletons have been reported: dolabellanes (including dolastanes), xenicanes, and extended sesquiterpenes [62].

##### Perhydroazulene Diterpenes

**Figure 11:**
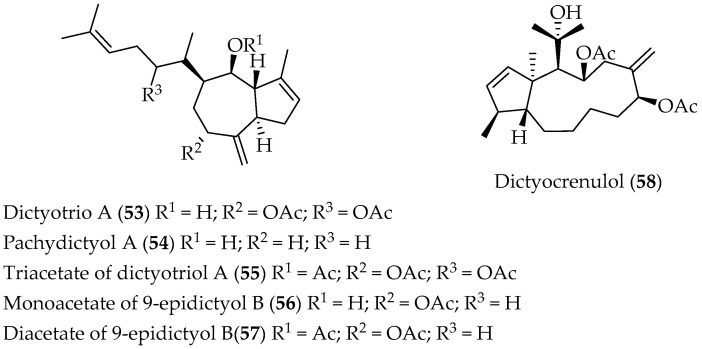
Perhydroazulene diterpenes isolated from the Chilean brown alga *Dictyota crenulata*.

Phytochemical study of brown algae *Dictyota crenulata*, collected at Vaihú, Easter Island, allowed chromatographic isolation of five diterpenes, **53**–**57** and **58** (Figure 11), whose chemical structures were determined by spectroscopic techniques [80,81].

Compounds **53** and **55** were tested against *Schizaphis graminum* and *Artemia salina*, and insecticidal activity against Tomato moth (*Tuta absolute*) was assayed as well [81].

##### Xenicane Diterpenes

**Figure 12:**
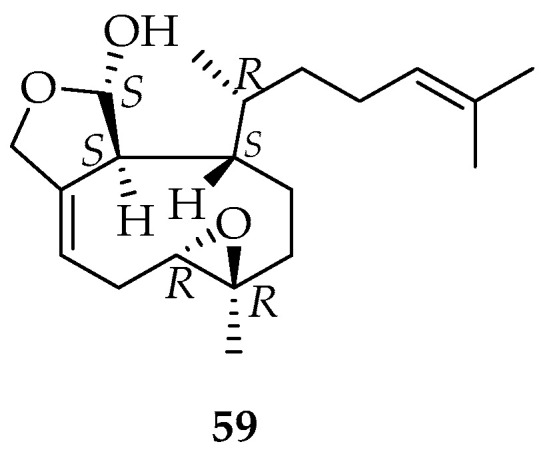
New xenicane diterpene isolated from the Chilean brown alga *Glossophora kunthii*.

A new diterpene with xenicane skeleton, **59** (Figure 12), has been obtained from *Glossophora kunthii*, collected at Valparaiso, Chile [82].

This new metabolite was fully characterized, including absolute stereochemistry. Other diterpenes, such as pachydictyol A and dictyotriol A C-12 monoacetate, were identified in this alga and their absolute configuration was determined by CD studies (exciton chirality method). These diterpenes are frequently isolated in the *Dictyota* genus [83].

##### Crenulides Diterpenes

**Figure 13:**
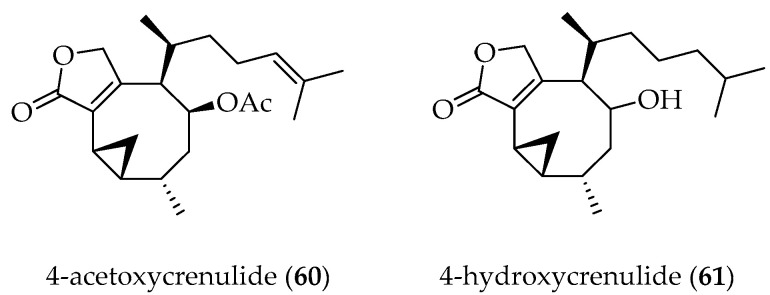
Crenulide diterpenes isolated from the Chilean brown alga *Glossophora kunthii*.

Two new crenulide diterpenes, **60** and **61** (Figure 13), have been obtained from the brown alga *G. kunthii*, collected at Horcones Bay, V Region, Chile. Their structures have been elucidated by spectral analysis and chemical correlation [84].

##### Plastoquinone Diterpenes

**Figure 14:**
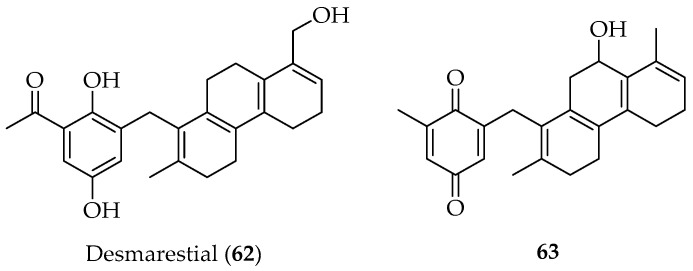
Plastoquinone diterpenes isolated from the Chilean brown alga *Desmarestia menziesii*.

Finally, two new plastoquinone diterpenes of mixed biogenesis, **62** and **63** (Figure 14), were isolated from the brown alga *Desmarestia menziesii*, collected near the Antarctic Peninsula (Chilean Base Arturo Prat) [85].

#### 2.1.5. Meroterpenoids

Meroterpenoids are prenylated aromatic compounds of mixed biogenesis combining acyclic, monocyclic, and bicyclic terpenes with aromatic or substituted aromatic groups possessing different degrees of oxidation. Many meroterpenoids from seaweeds have interesting biological activities such as antibacterial, antiviral, and antifeeding properties [62]. Plants in the genera Humulus and Cannabis produce these metabolites [86].

**Figure 15:**
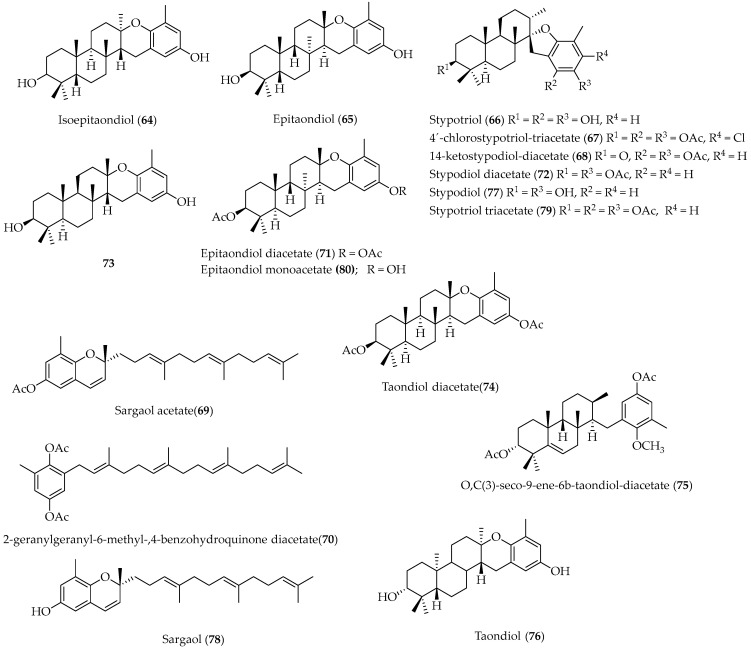
Meroterpenoids isolated from Chilean brown alga *Stypopodium flabelliforme*.

Seventeen meroterpenoids, **64**–**80** (Figure 15), were isolated from alga Stypopodium flabelliforme, collected at Easter Island [87,88,89,90,91,92]. The epitaondiol (**65**) structure was fully revised, and a meroditerpenoid containing an unusual two fused six-membered rings forced into the twist-boat conformation was demonstrated [87].

Structural elucidation of **67** and **68** (Figure 15) was carried out through spectroscopic analysis and theoretical studies. Remarkably, **67** was the first metabolite isolated from the *Stypopodium* genus that presents one halogen in its structure. At this point, it is worth noting that based on published NMR data of isoepitaondiol (**64**) (Figure 15), it was observed that the structure of compound **64** was wrongly assigned, and that the right structure of this compound corresponds to **65**. The relative configuration of this compound was confirmed by single-crystal X-ray diffraction, while the absolute configuration was evidenced by vibrational circular dichroism in combination with DFT B3LYP/DGDZVP calculations [91].

Taondiol (**76**) (Figure 15) has been isolated previously from this *Stypopodium* species. The absolute configurations of (−)-taondiol diacetate (**74**) and (+)-epitaondiol diacetate (**71**) isolated previously were determined using vibrational circular dichroism (VCD). For verification, their relative stereochemistry was determined by X-ray diffraction. Additionally, the crystal stereo structure of meroditerpenoid (**79**) (Figure 15) was reported [92].

Insecticidal activities of compounds **65**, **66** and **71** (Figure 15) were tested, mainly against *Spodoptera frugiperda*, and **71** showed the highest anti-insect activity. On the other hand, compound **71** showed no activity towards the National Cancer Institute’s test (U.S.A.) for agents active against HIV (killing of T lymphocytes by HIV) [88]. The inhibitory effects of **66** (Figure 15) were studied. Additionally, the molecular action of this compound on microtubule assembly was also analyzed [89,90].

The meroditerpenoids stypodiol (**77**), isoepitaondiol (**64**), and epitaondiol (**65**) exhibited gastroprotective activity in mice [93].

Compound **65** and sargaol (**78**) were tested on HCl/ethanol-induced gastric lesions in mice and compared with lansoprazole. Both **65** and **78** showed gastroprotective activity with ED_50_ values between 35 mg/kg and 40 mg/kg. The results suggest that **65** and **78** protect the gastric mucosa in the HCl/EtOH model in mice [94].

Pacifenol (**43**) (Figure 10), stypotriol triacetate (**79**) and epitaondiol (**65**) (Figure 15) were assayed for their anti-inflammatory effects. Compound **65** showed an important topical anti-inflammatory activity, whereas the other compounds showed a non-significant effect. Compound **65** inhibited human recombinant synovial phospholipase A_2_ activity in a concentration-dependent manner, whereas **40** effectively inhibited the degranulation response, but none of these compounds affected superoxide generation by human neutrophils [95].

Six meroditerpenoids, **65**, **68**, **71**, **77**, **79** and **80** (Figure 15), were tested for their cell proliferation inhibitory activity in five cell lines: Caco-2, SH-SY5Y, RBL-2H3, RAW.267 and V79. Overall, these compounds showed good activity against all cell lines, with SH-SY5Y and RAW.267 being the most susceptible. Their antimicrobial activity was also evaluated against *Enterococcus faecalis*, *Staphylococcus aureus*, *Proteus mirabilis*, *Salmonella typhimurium*, *Bacillus cereus*, and *Micrococcus luteus*. Antimicrobial capacity was observed for **77**, **79** and **80**, with the first being the most active [96].

### 2.2. C_15_-Acetogenins

Acetogenins are compounds biosynthesized from ethyl acetate or acetyl coenzyme A. Several halogenated C_15_-acetogenins, possessing acetylenes, allenes, and oxygen heterocycles, have been isolated from seaweeds [62]. Both linear and cyclic C_15_-acetogenins have been reported.

#### 2.2.1. Linear Polyhalogenated C_15_-Acetogenins

**Figure 16:**
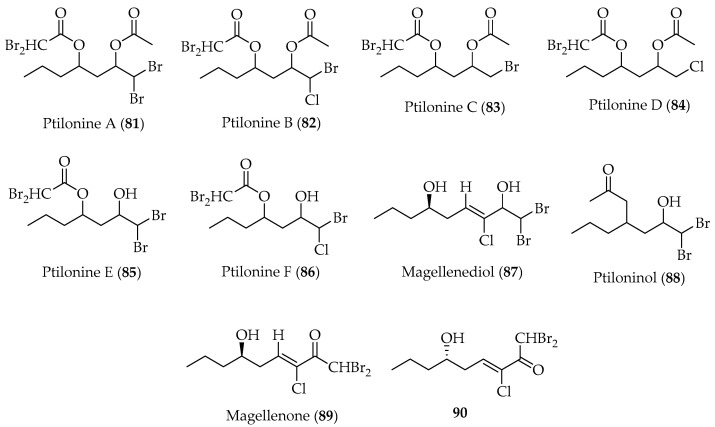
Linear polyhalogenated C_15_-acetogenins isolated from the Chilean marine alga *Ptilonia magellanica*.

Studies carried out on the red alga *Ptilonia magellanica*, collected around Fuerte Bulnes (Punta Arenas, XII region, Chile) at 3 m depth, led to isolation and identification of fifteen metabolites belonging to a single biosynthetic class [97,98]. Ptilonines A–F (**81**–**86**), magellenediol (**87**), magellenone (**88**) and ptiloninol (**89**) (Figure 16) are novel linear acetogenins that are described for the first time. Compounds **81**–**84** and **89** (Figure 16) were tested for their antimicrobial activity [97]. Results show that only compound **89** exhibited antibacterial activity against *K. pneumoniae* (MIC ≈ 100 mg/mL). It is worth noting that ptilonines present an unusual halogenation substitution pattern, which may confer evolutionary advantages to *P. magellanica*, for which a biogenetic origin is proposed [97].

#### 2.2.2. Cyclic Polyhalogenated C_15_-Acetogenins

**Figure 17:**
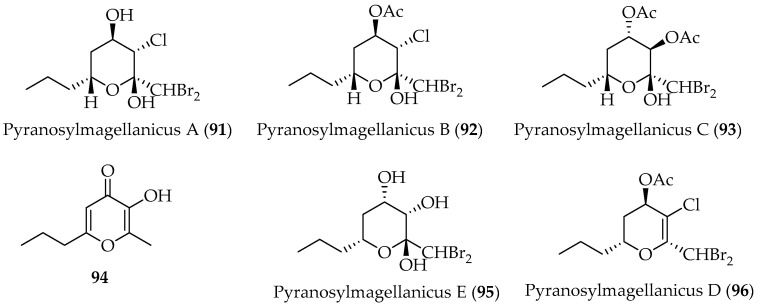
Cyclic polyhalogenated C_15_-acetogenins isolated from Chilean marine alga *Ptilonia magellanica.*

Previously reported γ-pyrone (**94**) [99] and five new cyclic polyhalogenated acetogenins, namely pyranosylmagellanicus A–C (**91**–**93**) and pyranosylmagellanicus D–E (**95** and **96**) (Figure 17), were obtained from the red alga *P. magellanica* [97,98]. These new metabolites are polyhalogenated pyranosyl-like hemiacetals that represent a novel structural type of acetogenin, being the first derivatives within the genus that incorporate chlorine in their structure [98]. These cyclic acetogenins present a common biosynthetic precursor, the linear acetogenin **90** (Figure 16).

The absolute configuration of the known pyranosylmagellanicus A (**91**), was determined by treatment of **91** with (R)- and (S)-α-methoxy-α-phenylacetic acids (MPA). Compounds **91**–**93** were tested for their antimicrobial activity, but no activity was found [97].

#### 2.2.3. Bromoallene C_15_-Acetogenins

**Figure 18:**
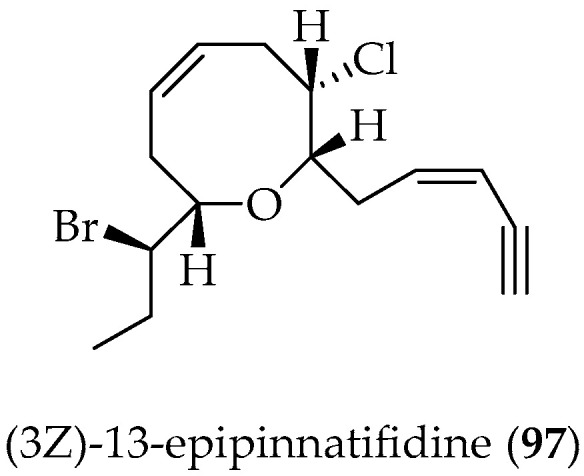
Bromoallene C_15_-acetogenins isolated from Chilean marine alga *Laurencia claviformis.*

Acetogenins that end in an enyne group are produced by algae from the genus Laurencia. Thus, (3Z)-13-epipinnatifidenyne (**97**) (Figure 18), a new C_15_-acetogenin, was obtained from the red alga *L. claviformis* collected at Easter Island. The structure of **97** was determined using 1D and 2D spectral analysis [100].

### 2.3. Furanones

2-Furanone is a heterocyclic organic compound. It is also known as γ-crotonolactone (GCL), as it is formally the lactone derived from γ-hydroxyisocrotonic acid. 4-Hydroxy-2,5-dimethyl-3(2H)-furanone (Furaneol^®^, HDMF, also 4-hydroxy-2,5-dimethyl-2,3-dihydrofuran-3-one) was identified for the first time in 1960, as a product of the Maillard reaction or nonenzymatic browning [101]. Previously, the synthesis and quorum sensing modulating effects of halogenated furanones isolated from the algae and their synthetic analogues have been reported [102].

**Figure 19:**
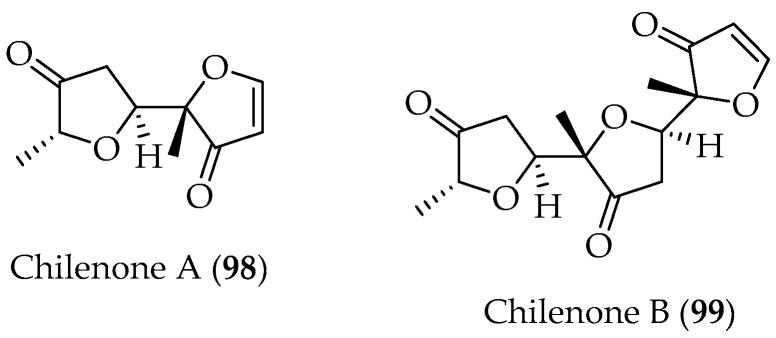
Furanones isolated from Chilean Marine Algae *Laurencia chilensis.*

Chilenone A (**98**) (Figure 19) was obtained from *Laurencia chilensis*, collected at Horcones Bay, Chile, and its structure was determined by spectroscopic and X-ray crystallographic techniques. The structure of **98** is unusual, and there are no previously reported antecedents in this respect. The potential precursor, 2-methyl-3(2β)-furanone has been known since 1929, but it has not been reported previously from any natural sources [103]. Chilenone B (**99**) (Figure 19) was obtained from a posterior collection of the same algae and its structure was established by X-ray diffraction and spectroscopic experiments. Compound **99** was identified as a trimer of 2-methyl-3(2H)-furanone [104].

**Figure 20:**
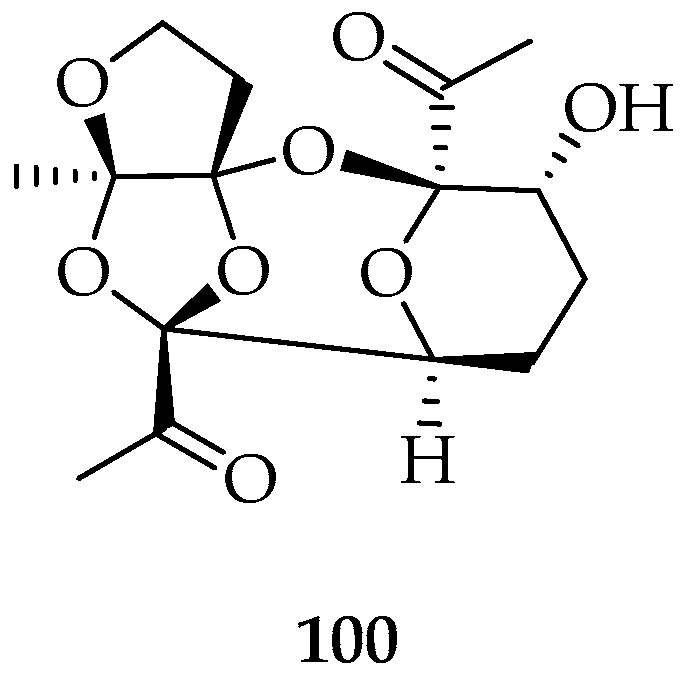
Tetracyclic polyketal isolated from Chilean marine alga *Laurencia chilensis.*

Finally, a new tetracyclic polyketal **100** (Figure 20) was identified from the marine red alga *L. chilensis*, collected in Cocholgüe, Concepción Bay, VIII region, Chile. This compound was isolated from chloroform extract, recrystallized from ethyl acetate and finally characterized by X-ray diffraction [105].

## 3. Conclusions

A first review of investigations carried out with marine algae from the Chilean coasts was published in 1989. Herein, we have updated the information by compiling all works, published up to mid-2019, on natural products isolated from algae collected along the coasts of Chile. The main species of algae that have been studied are *Plocamium cartilagineum*, *Shottera nicaensis*, *Pantoneura plocamioides*, *Ceramium rubrum*, *Laurencia claviformis*, *Laurencia chilensis*, *Dictyota crenulata*, *Glossophora kunthii*, *Desmarestia menziesii*, *Stypopodium flabelliforme*, and *Ptilonia magellanica*. From the total number of marine natural products identified (**92**), 31 are monoterpenes (**8**–**38**), 14 sesquiterpenes (**39**–**52**), 11 diterpenes (**53**–**63**), 17 meroterpenoids (**64**–**80**), 16 acetogenins (**81**–**89** and **91**–**97**), and three furanones (**98**–**100**). Among the biological activities studied in these compounds are the following: insecticidal activity of **27** and **28** against *Aphis fabae*, **53** and **55** against tomato moth (*Tuta absolute*), **65** against *Spodoptera frugiperda*; cytotoxicity of **31** against colon and cervical adenocarcinoma cells; inhibition of cytokinesis by **43** against *Tetrapygus niger*; toxicity against *Schizaphis graminum*; gastroprotective activity of **65** and **78** in mice; topical anti-inflammatory activity of **65** related to inhibition of leukocyte accumulation and human recombinant synovial phospholipase A_2_ activity; and inhibition of degranulation response by **40**. Compounds **65**, **68**, **71**, **77**, **79** and **80** were tested for their cell proliferation inhibitory activity in five cell lines: Caco-2, SH-SY5Y, RBL-2H3, RAW.267 and V79. Antimicrobial activity by **77**, **79** and **80**, and antibacterial activity by **89** against *K. pneumoniae* were determined.

The situation observed in the genus *Plocamium* is striking. The characteristic metabolites of this genus have been isolated from algae of the genera *Microcladia, Shotera, Pantoneura* and *Ceramium.* All of them belong to different families. So far, no explanation has been given for this phenomenon. On the other hand, it has been proposed that in *P. violaceum,* there would be two chemotypes depending on whether the monoterpenes are cyclic or lineal. An analogous situation can be described for *P. cartilagineum*; however, the description of oxygenated monoterpenes suggests that a possible chemotaxonomy of this species could become even more confusing.

Thus, from a chemical point of view, Chilean algae are characterized by the unique structures of some of their metabolites. However, considering the great variety of Chilean alga species, the number of works that have been published is relatively scarce. The extensive Chilean coasts are bathed by the cold Humboldt current, resulting in very cold waters in the extreme south and much warmer waters in the extreme north. Easter Island is a special case because it is in the middle of the ocean, far from the influence of this current and from any other kind of external influence. For these reasons, the marine biodiversity of the Chilean coast can be considered as an important source of new bioactive marine natural products that could be the basis for the development of new drugs but that have been poorly studied and exploited to date.
